# Comparison of Treatment Response, Survival Profiles, as Well as Safety Profiles Between CalliSpheres^®^ Microsphere Transarterial Chemoembolization and Conventional Transarterial Chemoembolization in Huge Hepatocellular Carcinoma

**DOI:** 10.3389/fonc.2021.793581

**Published:** 2022-01-21

**Authors:** Xuhua Duan, Juanfang Liu, Xinwei Han, Jianzhuang Ren, Hao Li, Fengyao Li, Shuguang Ju

**Affiliations:** Department of Interventional Radiology, The First Affiliated Hospital of Zhengzhou University, Zhengzhou, China

**Keywords:** huge HCC, DEB-TACE, treatment response, survival, safety

## Abstract

**Purpose:**

CalliSpheres^®^ microspheres (CSM) are the first drug-eluting beads (DEB) developed in China. This study aimed to compare treatment response, survival, and safety profiles between DEB transarterial chemoembolization (DEB-TACE) with CSM and conventional TACE (cTACE) in huge hepatocellular carcinoma (HCC) patients.

**Methods:**

A total of 71 patients with huge HCC who underwent DEB-TACE or cTACE were consecutively enrolled in this retrospective cohort study. Treatment response was assessed at first month (M1), third month (M3), and sixth month (M6) after TACE therapy; progression-free survival (PFS) and overall survival (OS) were evaluated; liver function indexes were recorded before TACE operation (M0), at first week (W1), M1 and M6 after TACE therapy; adverse events which occurred after TACE operation were recorded.

**Results:**

DEB-TACE presented with higher objective response rate (60.0% vs. 29.7%, *p* < 0.05) and disease control rate (86.7% vs. 59.4%, *p* < 0.05) compared with cTACE at M3. Regarding survival profiles, PFS [median: 3.3 months (95% CI: 2.8–3.7) vs. 2.1 months (95% CI: 1.7–2.5)] as well as OS [median: 7.8 months (95% CI: 4.6–11.0) vs. 5.7 months (95% CI: 5.0–6.3)] were longer in DEB-TACE group compared with cTACE group (both *p* < 0.01). Multivariate Cox’s regression further illustrated that DEB-TACE vs. cTACE was an independent protective factor for PFS and OS (both *p* < 0.01). As for safety profiles, patients’ liver function injury was reduced in the DEB-TACE group compared with the cTACE group. The incidence of fever was lower, and CINV was less severe in the DEB-TACE group compared with the cTACE group (both *p* < 0.05), while no difference in occurrence of liver abscess, increase of ascites, or moderate pain between two groups was observed.

**Conclusion:**

DEB-TACE with CSM presents with better treatment response, survival profiles, as well as safety profiles compared with cTACE in treatment for huge HCC patients.

## Introduction

Hepatocellular carcinoma (HCC) is the most common carcinoma as well as the second cause of cancer-related deaths in China, and huge HCC, accounting for around 20% of HCC cases, is defined as HCC whose nodule size is greater than 10 cm in diameter (1–3). Although the medical treatments for HCC have developed a lot in recent decades, there is still no recognized standard treatment for huge HCC, and the most commonly used curative approach for huge HCC is surgical resection, while due to large nodule size, high risk of tumor rupture, vascular invasion, as well as intrahepatic metastasis, the incidence of intraoperative death remains higher and postoperational survival is still worse in huge HCC patients compared with smaller HCC patients (4–6). Moreover, surgical resection is not applicable in most of HCC patients due to hidden onset of diseases, loss of liver function, and severe complications especially in huge HCC patients who need to resect over 80% of liver (7). Other therapeutic approaches such as associating liver partition and portal vein ligation for staged hepatectomy (ALPPS) and radiofrequency ablation are less commonly applied for treatment of huge HCC and are complicated with severe liver injury and risk of future liver remnant hypertrophy (8, 9). Therefore, exploring other nonsurgical therapeutic methods that both reduce the tumor size and control disease progression is necessary for treating huge HCC patients.

As one of the nonsurgical therapeutic methods, transarterial chemoembolization (TACE) is the most commonly used treatment for unresectable HCC (10, 11). Conventional TACE (cTACE) uses lipiodol as drug carrier to load and release the anticancer drugs, as well as gelatin sponge as embolization agents to block the blood supply to the targeted tumor, which has achieved generally good efficacy and safety profiles in treatment of huge HCC (12). However, cTACE poses high risk of systemic drug toxicity due to poor drug loading and releasing profiles as well as infixation of agents; therefore, drug-eluting microspheres, as new drug delivery and embolization agents for TACE, have been developed and realized sustained and optimized concentration of chemotherapy agents in tumor to overcome the limitations of cTACE (13, 14). For the application of drug-eluting bead TACE (DEB-TACE) in clinical settings, there are a number of studies disclosing favorable roles of DEB-TACE with various microspheres including CalliSpheres^®^ microspheres (CSM), DC^®^ beads, and HepaSpheres^®^ on treatment in general HCC patients compared with cTACE (15–22). CSM is the first DEB developed in China, which possesses good loading and releasing profile as well as acceptable biocompatibility; meanwhile, it exhibits good efficacy and tolerance in treating HCC patients (15–18).

Thus, this retrospective cohort study aimed to further compare treatment response, survival, and safety profiles between DEB-TACE with CSM and cTACE in huge HCC patients.

## Methods

### Patients

Seventy-one patients with huge HCC who underwent DEB-TACE or cTACE therapy in the First Affiliated Hospital of Zhengzhou University between Jan 2016 and Dec 2017 were consecutively enrolled in this retrospective cohort study. Huge HCC was defined as nodule size greater than 10 cm in diameter (1–3). The inclusion criteria consisted of the following: (1) diagnosed as primary HCC according to the American Association for the Study of Liver Diseases (AASLD) guidelines; (2) single nodule or multiple fused nodules in diameter above 10 cm; (3) aged 18 to 75 years; (4) received DEB-TACE or cTACE treatment; and (5) medical records were completely preserved and accessible. The exclusion criteria were as follows: (1) secondary HCC; (2) patients who had a history of malignancies except for ≥10 cm HCC; (3) patients who previously received DEB-TACE and cTACE therapy in other hospital; (4) patients who switched treatment between DEB-TACE and cTACE; (5) patients who received radiofrequency ablation, microwave ablation, particle implantation, or other interventional therapies after DEB-TACE or cTACE treatment; and (6) patients without any response assessment data or follow-up data. Finally, 31 patients who underwent DEB-TACE therapy were included in the DEB-TACE group, and 40 patients who received cTACE treatment were included in the cTACE group. No cross treatments were performed during 6 months. The present study was approved by the Institutional Review Board of the First Affiliated Hospital of Zhengzhou University, and written informed consents were obtained from all the patients or their statutory guardians.

### Baseline Information Collection

Patients’ baseline features were collected from electronic medical records, which included age, gender, cause of cirrhosis, largest nodule size, tumor location, portal vein tumor thrombus (PVTT), intrahepatic metastasis, extrahepatic metastasis, Eastern Cooperative Oncology Group (ECOG) performance status, Barcelona Clinic Liver Cancer (BCLC) stage, Child-Pugh stage, model for end-stage liver disease (MELD) score, hepatic artery-portal venous fistula (HAPVF), ascites, and splenomegaly.

### Procedures of DEB-TACE

In the present study, the CSM (Jiangsu Hengrui Medicine Co., Ltd., Jiangsu Province, China) with diameters of 300–500 μm were used in the DEB-TACE procedure. Before DEB-TACE, the CSM were loaded with pirarubicin (THP) (60–80 mg) (Shenzhen Main Luck Pharmaceuticals Inc., China) according to the manufacturer’s directions, subsequently, the high concentration contrast agent was added into the CSM (loaded with THP) as 1:1 ratio, and then the mixture of contrast agent and CSM loaded with THP was kept still for 5 min for further use. After the completion of drug-loading process, DEB-TACE was conducted as follows: firstly, local anesthesia was performed, then tumor-supplying vessels were detected by digital subtraction angiography (DSA). After the tumor-supplying vessel was identified and selected, the femoral artery was punctured by Seldinger technique using microcatheter with diameter of 4 F or 5 F (Merit Maestro, Merit Medical System, Inc., USA). A total of 100 mg oxaliplatin (Jiangsu Hengrui Medicine Co., Ltd., China) was then injected into the tumor-supplying vessel within 30 min; subsequently, the mixture of CSM was injected at a speed of 1 ml/min until the flow of contrast agent stagnated. Five minutes later, the angiography was performed again, and if there was incomplete embolization, DEB-TACE was performed for another time using Embospheres^®^ (Mai Ruitong Medical Devices Beijing Co., Ltd., China) with diameters of 300–500 μm.

### Procedures of cTACE

Suspension of 10 ml ethiodized poppyseed oil injection (EPO) (Jiangsu Hengrui Medicine Co., Ltd., China) and 20 mg THP was confected before cTACE. The processes of angiography and puncture of cTACE were performed as the same as DEB-TACE procedures, and the injection of oxaliplatin was also carried out as described above. After that, the suspension of 10 ml EPO and 20 mg THP was injected into the tumor-supplying vessel. If 10 ml EPO was not enough for complete embolization, gelatin sponge particles with diameters of 350–560 μm (Hangzhou Aili Pharmaceutical Technology Co., Ltd., China) were added until the stenosis of the flow occurred. In addition, the angiography was performed for another time to detect if there was incomplete embolization.

### Pain Management During and After TACE Operation

Analgesics were prepared before operation, which consisted of dexmedetomidine (Jiangsu Hengrui Medicine Co., Ltd., China), dezocin (Yangzi River Pharmaceutical Company, China), and 0.9% sodium chloride injection. At 30 min before the initiation of the operation, the analgesics were administrated to patients by patient-controlled analgesia (PCA), with the pump parameters as follows: the maintenance dose: 2.0 ml/h; the locking time: 15 min; the single dosage: 1.0 ml. If the pain was not tolerable, a single dosage could be added by pressing the single-dosage button. After operation, dezocine 5 mg + 2 ml 0.9% sodium chloride were given to the patients by intravenous injection if necessary (according to pain VAS score, if the score equal or above 7). Furthermore, symptomatic treatments were performed for postoperative complications.

### Treatment and Assessment After TACE Operation

Cinobufotalin was administrated to patients for antitumor therapy after TACE operations, which was given as follows: 1.2 g tid orally, 14 days per cycle, repeated every 14 days, and patients received at least 2 cycles of cinobufotalin. Enhanced computerized tomography (CT) or enhanced magnetic resonance imaging (MRI) was performed for treatment response assessment at first month (M1), third month (M3), and sixth month (M6) after first cycle of DEB-TACE or cTACE therapy, and as for patients with deficient deposit of EPO, residual lesions, or recurrence, DEB-TACE or cTACE was repeated. Treatment response was assessed according to the modified Response Evaluation Criteria in Solid Tumors (mRECIST), which included: (1) complete response (CR): disappearance of any intratumoral arterial enhancement in all target lesions; (2) partial response (PR): at least a 30% decrease in the sum of diameters of viable (enhancement in the arterial phase) target lesions; (3) stable disease (SD): any cases that did not qualify either PR or progressive disease (PD); and (4) PD: an increase of at least 20% in the sum of the diameters of the viable (enhancing) target lesions. In addition, objective response rate (ORR) was defined as the percentage of patients who achieved CR or PR, and disease control rate (DCR) was defined as proportion of patients who achieved CR, PR, or SD.

### Follow-Up

Liver function indexes including total bilirubin (TBIL), alanine aminotransferase (ALT), aspartate aminotransferase (AST), and albumin (ALB) were measured before TACE operation (M0), at first week after first cycle of TACE operation (W1), M1, and M6. Moreover, adverse events which occurred after TACE treatments were recorded as well as including liver abscess, increase of ascites, fever, moderate pain (VAS ≥4), and chemotherapy-induced nausea and vomiting (CINV). All patients were followed up by hospitalization and phone calls, and median follow-up time was 6.1 months (range: 2.8–14.7). Progression-free survival (PFS) was defined as the duration from the time of first TACE operation to the time of disease progression or death. Overall survival (OS) was defined as the duration from the time of first TACE operation to the time of death.

### Statistical Analysis

Statistical analysis was performed using SPSS 22.0 statistical software (SPSS Inc., USA), and figures were made by GraphPad Prism 6.01 software (GraphPad Software Inc., USA). Count data were expressed as count (percentage); normally distributed continuous data were presented as mean ± standard deviation; and skewed distributed continuous data were described as median (25th–75th quantiles). Comparison between two groups was determined by Chi-square test, *t*-test, or Wilcoxon rank sum test. Survival analysis was performed using Kaplan-Meier method, and difference of survival profiles between two groups was determined by log-rank test. Univariate and multivariate Cox’s proportional hazards regression analyses were used to determine prognostic factors of PFS and OS, and the multivariate Cox’s proportional hazards regression was performed using forward stepwise (conditional LR) method. *p*-value <0.05 was considered significant, and the significant results were shown in boldface.

## Results

### Study Flow

**Figure 1:**
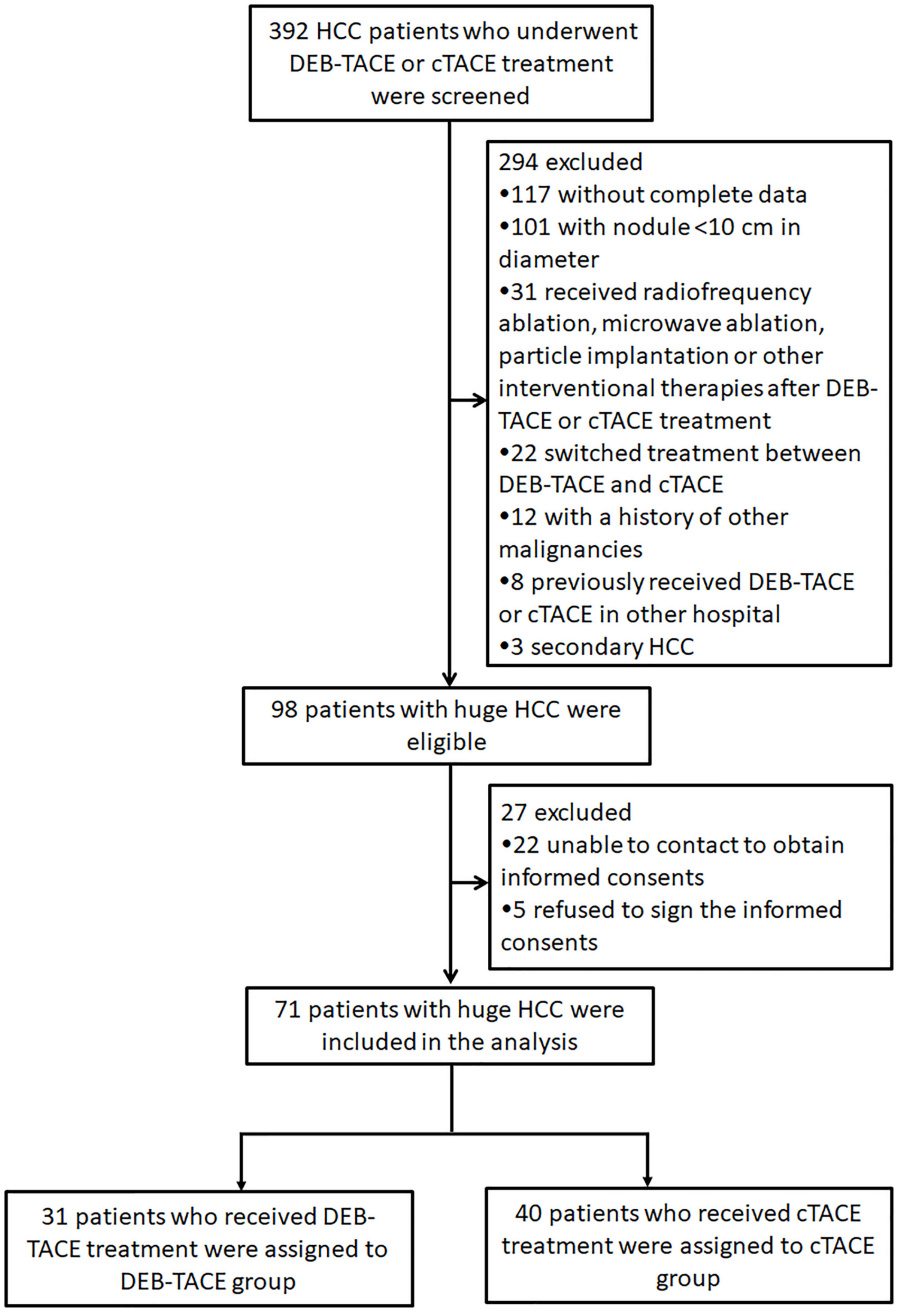
Study flow. HCC, hepatocellular carcinoma; DEB-TACE, drug-eluting bead transarterial chemoembolization; cTACE, conventional transarterial chemoembolization.

A total of 392 HCC patients who underwent DEB-TACE or cTACE treatment were initially screened, while 294 patients were excluded including 117 patients who were without complete data, 101 patients who were with nodule <10 cm in diameter, 31 patients who received radiofrequency ablation, microwave ablation, or other interventional therapies after DEB-TACE or cTACE treatment, 22 patients who switched treatment between DEB-TACE and cTACE, 12 patients who were with a history of other malignancies, 8 patients who previously received DEB-TACE or cTACE in other hospital, and 3 patients who had secondary HCC (**Figure 1**). Subsequently, 98 patients with huge HCC were eligible, whereas 27 of them were excluded including 22 patients who were unable to contact to obtain informed consents and 5 patients who refused to sign the informed consents. The remaining 71 patients with huge HCC were eventually included in the analysis. A total of 31 patients who received DEB-TACE were assigned to the DEB-TACE group and 40 patients who received cTACE were assigned to the cTACE group.

### Patients’ Baseline Characteristics

Huge HCC patients in DEB-TACE (*N* = 31) and cTACE (*N* = 40) groups were age and gender matched (**Table 1**). The mean age was 52.7 ± 9.4 years in the DEB-TACE group and 54.2 ± 11.2 years in the cTACE group (*p* = 0.557). There were 29 males and 2 females in the DEB-TACE group, while 33 males and 7 females in the cTACE group (*p* = 0.304). As for other baseline characteristics, no difference was observed between DEB-TACE and cTACE groups either regarding to cause of cirrhosis (*p* = 0.452), largest nodule size (*p* = 0.205), tumor location (*p* = 0.918), PVTT (*p* = 0.214), intrahepatic metastasis (*p* = 0.994), extrahepatic metastasis (*p* = 1.000), ECOG performance status (*p* = 0.826), BCLC stage (*p* = 0.639), Child-Pugh stage (*p* = 0.824), MELD score (*p* = 0.303), HAPVF (*p* = 0.747), ascites (*p* = 0.873), and splenomegaly (*p* = 0.306). In addition, the treatment cycles were 2.03 ± 0.98 times in the DEB-TACE group while 2.48 ± 0.78 times in the cTACE group (*p* = 0.035).

**Table 1:** Baseline characteristics.

Parameters	DEB-TACE group (*N* = 31)	cTACE group (*N* = 40)	*p*-value
Age (years)	52.7 ± 9.4	54.2 ± 11.2	0.557
Gender (male/female)	29/2	33/7	0.304
Cause of cirrhosis (*n*/%)			
Hepatitis B	21 (67.7)	29 (72.5)	0.452
Hepatitis C	2 (6.5)	4 (10.0)	
Alcohol	3 (9.7)	5 (12.5)	
Others	5 (16.1)	2 (5.0)	
Largest nodule size (cm)	10.7 (10.1–12.4)	11.6 (10.5–12.7)	0.205
Tumor location (*n*/%)			
Left liver	2 (6.5)	4 (10.0)	0.918
Right liver	29 (93.5)	36 (90.0)	
PVTT (*n*/%)	17 (54.8)	16 (40.0)	0.214
Intrahepatic metastasis (*n*/%)	7 (22.6)	9 (22.5)	0.994
Extrahepatic metastasis (*n*/%)	5 (16.1)	6 (15.0)	1.000
ECOG performance status (*n*/%)			
0	2 (6.5)	4 (10.0)	0.826
1	10 (32.3)	13 (32.5)	
2	16 (51.6)	18 (45.0)	
3	3 (9.7)	5 (12.5)	
BCLC stage (*n*/%)			
B	7 (22.6)	11 (27.5)	0.639
C	24 (77.4)	29 (72.5)	
Child-Pugh stage (*n*/%)			
A	17 (54.8)	23 (57.5)	0.824
B	14 (45.2)	17 (42.5)	
MELD score	19.0 (17.0–21.0)	17.0 (16.0–23.8)	0.303
HAPVF (*n*/%)	6 (19.4)	9 (22.5)	0.747
Ascites (*n*/%)	8 (25.8)	11 (27.5)	0.873
Splenomegaly (*n*/%)	25 (80.6)	28 (70.0)	0.306
Current TACE cycles	2.03 ± 0.98	2.48 ± 0.78	**0.035**

Data were presented as mean value ± standard deviation, count (percentage), or median (25th–75th quantiles). Comparison was determined by t-test, Chi-square test, or Wilcoxon rank sum test. p-value < 0.05 was considered significant (in bold). DEB-TACE, drug-eluting bead transarterial chemoembolization; cTACE, conventional transarterial chemoembolization; PVTT, portal vein tumor thrombus; ECOG, Eastern Cooperative Oncology Group; BCLC, Barcelona Clinic Liver Cancer; MELD, model for end-stage liver disease; HAPVF, hepatic artery-portal venous fistula.

### Comparison of Treatment Response Rate Between the DEB-TACE and cTACE Groups

**Figure 2:**
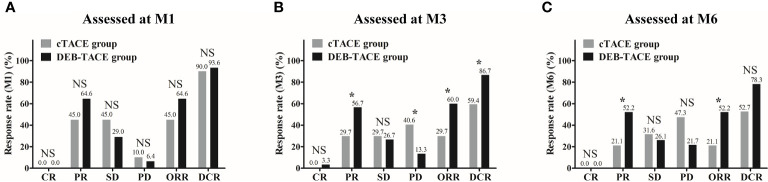
Treatment response rate between the DEB-TACE and cTACE groups. No difference in CR, ORR, or DCR was observed between the DEB-TACE and cTACE groups at M1 **(A)**. At M3, CR was similar, whereas ORR and DCR were higher in the DEB-TACE group compared with the cTACE group **(B)**. At M6, ORR was higher while CR and DCR were similar in the DEB-TACE group compared with the cTACE group **(C)**. Comparisons of response rates between the two groups were performed using Chi-square test, and *p* < 0.05 was considered significant. ^*^ *p* < 0.05; NS, not significant; DEB-TACE, drug-eluting bead transarterial chemoembolization; cTACE, conventional transarterial chemoembolization; CR, complete response; PR partial response; SD, stable disease; PD, progressive disease; ORR, objective response rate; DCR, disease control rate; M, month.

Comparison of treatment response rate between the DEB-TACE and cTACE groups was performed using Chi-square test. At M1 after treatment, no difference in CR, ORR, or DCR was observed between the two groups (All *p* > 0.05) (**Figure 2A**). At M3 after treatment, CR was similar (*p* > 0.05) but ORR (*p* < 0.05) and DCR (*p* < 0.05) were higher in the DEB-TACE group compared with the cTACE group (**Figure 2B**). At M6 after treatment, ORR was higher (*p* < 0.05) while CR (*p* > 0.05) and DCR (*p* > 0.05) were similar in the DEB-TACE group compared with the cTACE group (**Figure 2C**). These implied that DEB-TACE resulted in better treatment response in huge HCC patients compared with cTACE.

### Short-Term Mortality and Causes of Death Between the DEB-TACE and cTACE Groups

**Figure 3:**
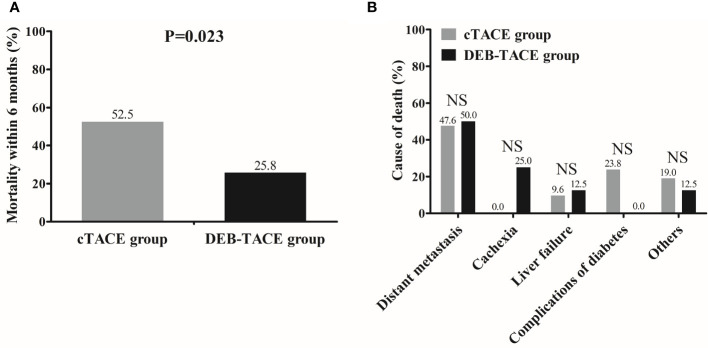
Mortality and death causes between the DEB-TACE and cTACE groups. Mortality within 6 months was lower in the DEB-TACE group compared with cTACE group **(A)**, and there was no difference in death causes between the two groups **(B)**. Comparison of mortality rate as well as death causes between two groups was determined by Chi-square test. *p* < 0.05 was considered significant. NS, not significant; DEB-TACE, drug-eluting bead transarterial chemoembolization; cTACE, conventional transarterial chemoembolization.

The 6-month mortality rate was 25.8% in the DEB-TACE group, which was decreased compared with that in the cTACE group (52.5%) (*p* = 0.023) (**Figure 3A**). The comparison of death causes revealed that no difference in distant metastasis, cachexia, liver failure, complications of diabetes, or other causes of death was observed between the DEB-TACE and cTACE groups (All *p* > 0.05) (**Figure 3B**).

### Comparison of PFS and OS Between the DEB-TACE and cTACE Groups

**Figure 4:**
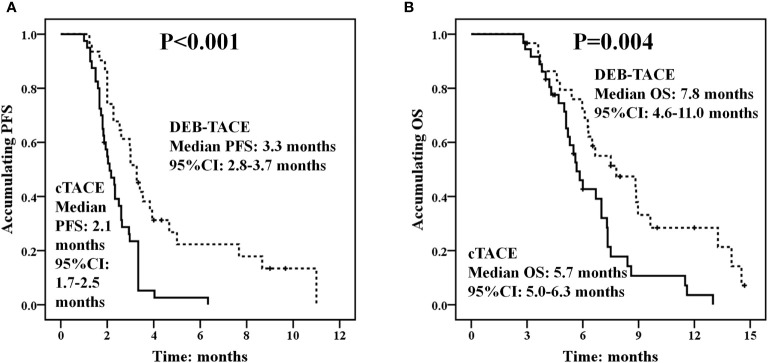
PFS and OS between the DEB-TACE and cTACE groups. PFS was better in the DEB-TACE group compared with the cTACE group **(A)**, and longer OS was observed in the DEB-TACE group compared with the cTACE group as well **(B)**. Kaplan-Meier method was used to assess PFS and OS of huge HCC patients and difference between the two groups was determined by log-rank test. *p* < 0.05 was considered significant. DEB-TACE, drug-eluting bead transarterial chemoembolization; cTACE, conventional transarterial chemoembolization; PFS, progression-free survival; OS, overall survival.

Kaplan-Meier method was used to assess PFS and OS of huge HCC patients and difference between the DEB-TACE and cTACE groups was determined by log-rank test. PFS was longer in the DEB-TACE group (median PFS: 3.3 months, 95% CI: 2.8–3.7 months) compared with the cTACE group (median PFS: 2.1 months, 95% CI: 1.7–2.5 months) (*p* < 0.001) (**Figure 4A**). Also, OS was increased in the DEB-TACE group (median OS: 7.8 months; 95% CI: 4.6–11.0 months) compared with the cTACE group (median OS: 5.7 months, 95% CI: 5.0–6.3 months) (*p* = 0.004) (**Figure 4B**).

### Factors Affecting PFS

Univariate Cox’s proportional hazards regression displayed that DEB-TACE vs. cTACE (*p* = 0.001) was correlated with longer PFS, while PVTT (*p* = 0.001), intrahepatic metastasis (*p* = 0.020), extrahepatic metastasis (*p* < 0.001), ECOG performance status (≥2 vs. <2) (*p* = 0.009), BCLC stage (C vs. B) (*p* = 0.005), Child-Pugh stage (B vs. A) (*p* = 0.009), and HAPVF (*p* < 0.001) were associated with shorter PFS (**Table 2**). Further multivariate Cox’s regression with forward stepwise (conditional LR) method revealed that DEB-TACE vs cTACE (*p* < 0.001) independently predicted better PFS, while extrahepatic metastasis (*p* < 0.001), BCLC stage (C vs. B) (HR = 3.205, *p* = 0.001), and HAPVF (*p* = 0.005) independently predicted worse PFS in huge HCC patients.

**Table 2:** Factors affecting PFS by Cox’s proportional hazards regression model analysis.

Parameters	Cox’s regression model			
	*p*-value	HR	95% CI	
			Lower	Higher
**Univariate Cox’s regression**				
DEB-TACE vs. cTACE	**0.001**	0.392	0.229	0.670
Age (≥54 vs. <54)	0.542	1.166	0.711	1.914
Gender (male vs. female)	0.960	0.982	0.482	1.999
Cause of cirrhosis				
Hepatitis B vs. others	0.266	1.373	0.785	2.401
Hepatitis C vs. others	0.673	0.821	0.328	2.054
Alcohol vs. others	0.858	0.931	0.424	2.045
Largest nodule size (≥11 cm vs. <11 cm)	0.416	1.228	0.748	2.016
Tumor location (right vs. left)	0.276	0.623	0.265	1.461
PVTT	**0.001**	2.259	1.374	3.712
Intrahepatic metastasis	**0.020**	1.986	1.116	3.535
Extrahepatic metastasis	**<0.001**	11.948	5.346	26.704
ECOG performance status (≥2 vs. <2)	**0.009**	1.979	1.187	3.298
BCLC stage (C vs. B)	**0.005**	2.344	1.287	4.267
Child-Pugh stage (B vs. A)	**0.009**	1.958	1.184	3.236
MELD score (≥18 vs. <18)	0.199	0.724	0.442	1.185
HAPVF	**<0.001**	4.343	2.271	8.307
Ascites	0.090	1.610	0.928	2.794
Splenomegaly	0.782	1.085	0.609	1.933
**Multivariate Cox’s regression with forward stepwise (conditional LR) method**				
DEB-TACE vs. cTACE	**<0.001**	0.192	0.106	0.350
Extrahepatic metastasis	**<0.001**	11.804	4.418	31.542
BCLC stage (C vs. B)	**0.001**	3.205	1.657	6.199
HAPVF	**0.005**	3.219	1.437	7.211

Factors affecting PFS were determined by univariate and multivariate Cox’s proportional hazards regression model analyses, and the multivariate Cox’s proportional hazards regression was performed with forward stepwise (conditional LR) method. p-value <0.05 was considered significant (in bold). PFS, progression-free survival; HR, hazards ratio; CI, confidence interval; DEB-TACE, drug-eluting bead transarterial chemoembolization; cTACE, conventional transarterial chemoembolization; PVTT, portal vein tumor thrombus; ECOG, Eastern Cooperative Oncology Group; BCLC, Barcelona Clinic Liver Cancer; MELD, model for end-stage liver disease; HAPVF, hepatic artery-portal venous fistula.

### Factors Affecting OS

For OS, univariate Cox’s proportional hazards regression disclosed that DEB-TACE vs. cTACE (*p* = 0.005) was associated with better OS, whereas PVTT (*p* < 0.001), intrahepatic metastasis (*p* = 0.018), extrahepatic metastasis (*p* < 0.001), ECOG performance status (≥2 vs. <2) (*p* = 0.017), BCLC stage (C vs. B) (*p* = 0.003), Child-Pugh stage (B vs. A) (*p* = 0.008), HAPVF (*p* < 0.001), and ascites (*p* = 0.003) were correlated with worse OS (**Table 3**). In addition, multivariate Cox’s regression with forward stepwise (conditional LR) method illustrated that DEB-TACE vs. cTACE (*p* < 0.001) independently predicted longer OS, while PVTT (*p* = 0.004), intrahepatic metastasis (*p* < 0.001), extrahepatic metastasis (*p* = 0.024), ECOG performance status (≥2 vs. <2) (*p* < 0.001), and HAPVF (*p* < 0.001) independently predicted shorter OS in huge HCC patients.

**Table 3:** Factors affecting OS by Cox’s proportional hazards regression model analysis.

Parameters	Cox’s regression model			
	*p*-value	HR	95% CI	
			Lower	Higher
**Univariate Cox’s regression**				
DEB-TACE vs. cTACE	**0.005**	0.434	0.242	0.778
Age (≥54 vs. <54)	0.590	1.163	0.671	2.017
Gender (male vs. female)	0.688	1.179	0.527	2.638
Cause of cirrhosis				
Hepatitis B vs. others	0.128	1.642	0.866	3.113
Hepatitis C vs. others	0.790	0.880	0.341	2.267
Alcohol vs. others	0.579	0.783	0.330	1.859
Largest nodule size (≥11 cm vs. <11 cm)	0.444	1.241	0.714	2.158
Tumor location (right vs. left)	0.343	0.636	0.249	1.622
PVTT	**<0.001**	3.225	1.829	5.685
Intrahepatic metastasis	**0.018**	2.224	1.150	4.303
Extrahepatic metastasis	**<0.001**	23.061	7.650	69.517
ECOG performance status (≥2 vs. <2)	**0.017**	1.979	1.130	3.465
BCLC stage (C vs. B)	**0.003**	2.700	1.406	5.186
Child-Pugh stage (B vs. A)	**0.008**	2.159	1.224	3.808
MELD score (≥18 vs. <18)	0.585	0.859	0.497	1.483
HAPVF	**<0.001**	7.948	3.754	16.828
Ascites	**0.003**	2.570	1.391	4.749
Splenomegaly	0.888	1.045	0.562	1.943
**Multivariate Cox’s regression with forward stepwise (conditional LR) method**				
DEB-TACE vs. cTACE	**<0.001**	0.156	0.073	0.334
PVTT	**0.004**	2.896	1.394	6.017
Intrahepatic metastasis	**<0.001**	6.332	2.823	14.200
Extrahepatic metastasis	**0.024**	5.664	1.258	25.500
ECOG performance status (≥2 vs. <2)	**<0.001**	5.297	2.346	11.958
HAPVF	**<0.001**	15.420	4.758	49.979

Factors affecting OS were determined by univariate and multivariate Cox’s proportional hazards regression model analyses, and the multivariate Cox’s proportional hazards regression was performed with forward stepwise (conditional LR) method. p-value < 0.05 was considered significant (in bold). OS, overall survival; HR, hazard ratio; CI, confidence interval; DEB-TACE, drug-eluting bead transarterial chemoembolization; cTACE, conventional transarterial chemoembolization; PVTT, portal vein tumor thrombus; ECOG, Eastern Cooperative Oncology Group; BCLC, Barcelona Clinic Liver Cancer; MELD, model for end-stage liver disease; HAPVF, hepatic artery-portal venous fistula.

### Comparison of Liver Function Indexes Between the DEB-TACE and cTACE Groups

No difference in liver function parameters was observed between the DEB-TACE and cTACE groups at M0, W1, or M1 (All *p* > 0.05), whereas at M6, TBIL (*p* = 0.045), ALT (*p* = 0.007), and AST (*p* = 0.047) were higher, but ALB (*p* < 0.001) was lower in the DEB-TACE group compared with the cTACE group (**Table 4**).

**Table 4:** Change of liver function before and after treatment.

Parameters	Time	DEB-TACE group (*N* = 31)	cTACE group (*N* = 40)	*p*-value
TBIL (μmol/L)	M0	41.9 ± 72.6	41.5 ± 90.2	0.984
	W1	42.4 ± 62.5	48.8 ± 97.4	0.752
	M1	34.9 ± 56.2	43.8 ± 81.7	0.589
	M6	63.8 ± 72.0	116.1 ± 77.6	**0.045**
ALT (U/L)	M0	61.9 ± 81.7	66.6 ± 75.3	0.802
	W1	85.8 ± 65.3	103.3 ± 103.4	0.415
	M1	36.8 ± 22.3	49.9 ± 35.5	0.063
	M6	63.5 ± 31.1	93.4 ± 30.8	**0.007**
AST (U/L)	M0	85.9 ± 78.5	69.0 ± 77.1	0.367
	W1	99.3 ± 94.3	90.7 ± 62.6	0.648
	M1	76.1 ± 47.3	72.5 ± 118	0.872
	M6	95.1 ± 34.8	137.4 ± 71.3	**0.047**
ALB (g/L)	M0	35.8 ± 4.2	35.2 ± 4.1	0.570
	W1	31.7 ± 4.6	31.4 ± 4.3	0.747
	M1	34.2 ± 4.2	33.4 ± 4.6	0.461
	M6	31.0 ± 3.3	26.5 ± 1.8	**<0.001**

Data were presented as mean value ± standard deviation. Comparison was determined by t-test. p-value < 0.05 was considered significant (in bold). DEB-TACE, drug-eluting bead transarterial chemoembolization; cTACE, conventional transarterial chemoembolization; TBIL, total bilirubin; ALT, alanine aminotransferase; AST, aspartate aminotransferase; ALB, albumin.

### Comparison of Adverse Events Between the DEB-TACE and cTACE Groups

Incidences of adverse events which occurred after TACE treatments were recorded, and comparisons of adverse events between the DEB-TACE and cTACE groups were performed (**Table 5**). The incidence of fever (*p* = 0.034) was lower, and CINV grade (*p* = 0.001) was less severe in the DEB-TACE group compared with the cTACE group, whereas no difference in occurrence of liver abscess (*p* = 1.000), increase of ascites (*p* = 1.000), or moderate pain (*p* = 0.946) was observed between the two groups.

**Table 5:** Adverse events.

Parameters	DEB-TACE group (*N* = 31)	cTACE group (*N* = 40)	*p*-value
Liver abscess (*n*/%)	0 (0.0)	1 (2.5)	1.000
Increase of ascites (*n*/%)	3 (9.7)	4 (10.0)	1.000
Fever (*n*/%)	10 (32.3)	23 (57.5)	**0.034**
Moderate pain (VAS ≥4) (*n*/%)	6 (19.4)	8 (20.0)	0.946
CINV grade (*n*/%)			
0	8 (25.8)	3 (7.5)	**0.001**
I	14 (45.2)	9 (22.5)	
II	6 (19.4)	17 (42.5)	
III	3 (9.7)	11 (27.5)	

Data were presented as count (percentage). Comparison was determined by Chi-square test or Wilcoxon rank sum test. p-value < 0.05 was considered significant (in bold). DEB-TACE, drug-eluting bead transarterial chemoembolization; cTACE, conventional transarterial chemoembolization; VAS, visual analogue scale; CINV, chemotherapy-induced nausea and vomiting.

## Discussion

Our results disclosed that in huge HCC patients: (1) DEB-TACE with CSM yielded better treatment response compared with cTACE. (2) Short-term mortality rate (within 6 months) was lower, and PFS as well as OS were longer in the DEB-TACE group compared with the cTACE group. (3) Compared with cTACE, DEB-TACE resulted in decreased level of liver function injury at M6 as well as lower incidence of adverse events after TACE treatments.

Treatment is even harder and prognosis is worse for huge HCC compared with smaller HCC due to advanced and complex disease conditions, and there is no consensus on a standard treatment strategy for huge HCC (4). Currently, surgical resection is recognized as curative therapy for huge HCC, while the success rate of surgery is much lower in huge HCC compared with smaller HCC, and the postsurgical survival is also unsatisfactory (7). Also, ALPPS is applied for treatment of huge HCC, whereas it induces extensive future liver remnant hypertrophy and high morbidity (8). Other therapeutic approaches such as high-intensity-focused ultrasound and radiofrequency ablation are increasingly investigated for huge HCC management, while their treatment outcomes are limited by complicated tumor location as well as severe complications in huge HCC (8, 9, 23). Apart from these, cTACE has been illustrated by accumulating studies to present relatively good treatment efficacy in treating huge HCC, whereas for DEB-TACE, which overcomes several limitations of cTACE and has been reported to achieve better treatment outcomes compared with cTACE in general HCC patients, its roles in huge HCC patients are still obscure (10, 13, 14, 24, 25). Therefore, in this study, we assumed that for huge HCC patients, DEB-TACE might also possess satisfying treatment outcomes, and compared the efficacy, survival profiles, as well as safety profiles between DEB-TACE with CSM and cTACE in huge HCC patients.

Drug-eluting microspheres are developed for loading and slowly releasing cytotoxic drugs into the tumor, and they also act as embolization agents to block blood supply to hypervascular tumors (26). DEB-TACE using these microspheres have been extensively investigated regarding treatment response in HCC patients. For instance, a study in China exhibits that DEB-TACE using CSM results in better ORR compared with cTACE at the second cycle of treatment in HCC patients at BCLC stage C (17). In addition, short-term (3–6 months) ORR and DCR are higher in the DEB-TACE with CSM group compared with the cTACE group in general HCC patients (19). Also, DEB-TACE with DC^®^ beads presents better treatment response compared with cTACE in HCC patients (27). These previous studies imply that DEB-TACE performs better than cTACE in treatment response in general HCC patients, whereas the roles of DEB-TACE on huge HCC patients are less investigated. Therefore, we evaluated treatment response to DEB-TACE and cTACE in huge HCC patients and discovered that ORR and DCR were higher in the DEB-TACE group compared with the cTACE group. The possible explanations were as follows: (1) microspheres had better drug loading and releasing profiles than lipiodol used in cTACE, resulting in higher concentration of drug at targeted tumor, thereby more effectively killing cancer cells and inducing tumor necrosis compared with cTACE. Meanwhile, due to homogeneity of the microspheres and vascular deformability, the peripheral embolization effect of DEB-TACE was better; therefore, DEB-TACE presented better treatment response compared with cTACE. (2) Regarding stability of treatment response, the drug concentration at targeted tumors might be more stable and slowly reduced in DEB-TACE due to constant release of drugs compared with cTACE; therefore, DEB-TACE achieved more sustained treatment response compared with cTACE.

As for survival profiles of DEB-TACE and cTACE in HCC patients, discrepancy still exists in different clinical researches. Some studies state that DEB-TACE is better than cTACE in prolonging patients’ survival, whereas other voices claim that there is no difference in survival profiles between these two treatments (28–31). Considering that these previous studies focus on general HCC patients, and information of DEB-TACE in huge HCC is limited, we compared survival profiles between DEB-TACE and cTACE in huge HCC patients and discovered that short-term mortality rate was lower, and PFS as well as OS were longer in the DEB-TACE group compared with the cTACE group. These could be due to that: (1) benefiting from the drug loading and releasing profiles of microspheres, DEB-TACE was illustrated to achieve and sustain optimal drug concentration as well as retain treatment response for a longer duration compared to cTACE, therefore, patients’ survival profiles were better in DEB-TACE group compared to cTACE group (32). (2) DEB-TACE might lead to less escape of drugs to normal liver tissue and adjacent organs, reducing the risk of liver function injury as well as the systemic cytotoxicity, thereby favoring survival profiles in huge HCC patients. Moreover, we performed multivariate Cox’s regression analysis to further screen factors that independently affected survivals of huge HCC patients and discovered that DEB-TACE vs. cTACE was an independent protective factor for PFS and OS, which again supported our results that DEB-TACE yielded better survival profiles compared with cTACE.

It was worth noting that cinobufotalin was used following TACE treatment in our study. Cinobufotalin serves a main cardiac toxin in toad, which is also reported to be a novel anti-HCC agent: it facilitates tumor growth inhibition and induces apoptosis in cultured HCC cells *via* ceramide production (33, 34). Meanwhile, cinobufotalin is also uncovered to reverse multidrug resistance of tumors including HCC, such as it can reverse the adriamycin resistance in Raji/ADR cells and the expression of P-gp and MRP-1 protein (33, 34). It is also recommended for HCC treatment in clinical practice (35). Therefore, it was applied in our study.

Although TACE has achieved promising efficacy in treatment for HCC, it is also illustrated to cause embolic syndrome (with incidence exceeding 10%) including liver dysfunction, pain, ascites, and CINV due to embolization of the blood-transferring arteries (36). In general HCC patients, safety profiles have been investigated between DEB-TACE and cTACE based on the symptoms of embolic syndrome. For example, a recent study comparing the short-term safety elucidates that liver function is better reserved and the incidence of drug-related complications is lower in the DEB-TACE group with CSM compared with the cTACE group (19). A randomized controlled trial reveals that patients receiving DEB-TACE with DC^®^ beads experience less procedural abdominal pain than those treated with cTACE (31). Additionally, the ALT change from baseline to 48 h after TACE procedure is decreased indicating less liver function injury in patients underwent DEB-TACE with DC^®^ beads compared with cTACE (27). These previous studies imply that DEB-TACE is relatively safe and well tolerated in treatment of general HCC patients, whereas for huge HCC patients, its safety profiles still remain unclear. In line with the previous studies, our study observed that hepatic injury was less and the incidence of adverse events was lower in the DEB-TACE group compared with the cTACE group in huge HCC patients. These could be explained by that EPO that was used in cTACE might lead to fast escape and metabolization of cytotoxic drugs, hence increased the toxicity to normal liver tissues and adjacent organs, thereby aggravating liver function injury and adverse events in huge HCC patients. Whereas for DEB-TACE, it achieved stable and sustained release of drugs to the targeted tumor and less drug escape to the adjacent tissues, which reduced systemic drug toxicity, and presented with less liver function injury and lower incidence of adverse events.

There were still several limitations in our study: (1) As a retrospective study with relatively small sample size, the statistical power of our results might be mitigated; meanwhile, it was not a randomized design; therefore, further studies preferably randomized controlled trials or prospective studies with larger sample size were needed to verify the results. (2) Analgesics were administered during and after treatment on requirement. Therefore, considering that patients might have different tolerance degree to pain and received different dose of analgesics, the result regarding pain in adverse events might be influenced. (3) Embospheres^®^ (with diameters of 300–500 μm) was used if embolization was not complete, which might become a cofounding factor for treatment outcomes. (4) The follow-up duration in this study was relatively short, therefore comparison of long-term efficacy between DEB-TACE and cTACE on treatment outcomes in huge HCC patients were not investigated.

In conclusion, DEB-TACE with CSM presents with better treatment response, survival profiles, as well as safety profiles compared with cTACE in the treatment for huge HCC patients.

## Data Availability Statement

The original contributions presented in the study are included in the article/supplementary material. Further inquiries can be directed to the corresponding authors.

## Ethics Statement

The studies involving human participants were reviewed and approved by the First Affiliated Hospital of Zhengzhou University. The patients/participants provided their written informed consent to participate in this study.

## Author Contributions

XH and JR contributed to the conception. XD, JL, HL, and FL contributed to data acquisition and data analysis. SJ, XD, and JL drafted the manuscript. XH and JR revised the manuscript. All authors read and approved the final manuscript.

## Funding

This work was supported by the National Natural Science Foundation of China (No. 81401494).

## Conflict of Interest

The authors declare that the research was conducted in the absence of any commercial or financial relationships that could be construed as a potential conflict of interest.

## Publisher’s Note

All claims expressed in this article are solely those of the authors and do not necessarily represent those of their affiliated organizations, or those of the publisher, the editors and the reviewers. Any product that may be evaluated in this article, or claim that may be made by its manufacturer, is not guaranteed or endorsed by the publisher.
